# Reconstructing hotspots of genetic diversity from glacial refugia and subsequent dispersal in Italian common toads (*Bufo bufo*)

**DOI:** 10.1038/s41598-020-79046-y

**Published:** 2021-01-08

**Authors:** Andrea Chiocchio, Jan. W. Arntzen, Iñigo Martínez-Solano, Wouter de Vries, Roberta Bisconti, Alice Pezzarossa, Luigi Maiorano, Daniele Canestrelli

**Affiliations:** 1 Department of Ecological and Biological Science, Tuscia University, Largo dell’Università s.n.c., 01100 Viterbo, Italy; 2 Naturalis Biodiversity Center, P.O. Box 9517, 2300 RA Leiden, The Netherlands; 3 Department of Biodiversity and Evolutionary Biology, Museo Nacional de Ciencias Naturales, CSIC, c/ José Gutiérrez Abascal 2, 28006 Madrid, Spain; 4 Asociation Ambor, Ctra. Constantina – Pedroso 1, 41450 Constantina, Spain; 5 Department of Biology and Biotechnology “Charles Darwin”, Università di Roma La Sapienza, Viale dell’Università 32, 00185 Rome, Italy

**Keywords:** Ecology, Evolution

## Abstract

Genetic diversity feeds the evolutionary process and allows populations to adapt to environmental changes. However, we still lack a thorough understanding of why hotspots of genetic diversity are so 'hot'. Here, we analysed the relative contribution of bioclimatic stability and genetic admixture between divergent lineages in shaping spatial patterns of genetic diversity in the common toad *Bufo bufo* along the Italian peninsula. We combined population genetic, phylogeographic and species distribution modelling (SDM) approaches to map ancestral areas, glacial refugia, and secondary contact zones. We consistently identified three phylogeographic lineages, distributed in northern, central and southern Italy. These lineages expanded from their ancestral areas and established secondary contact zones, before the last interglacial. SDM identified widespread glacial refugia in peninsular Italy, sometimes located under the present-day sea-level. Generalized linear models indicated genetic admixture as the only significant predictor of the levels of population genetic diversity. Our results show that glacial refugia contributed to preserving both levels and patterns of genetic diversity across glacial-interglacial cycles, but not to their formation, and highlight a general principle emerging in Mediterranean species: higher levels of genetic diversity mark populations with substantial contributions from multiple genetic lineages, irrespective of the location of glacial refugia.

## Introduction

Intraspecific genetic variation feeds the evolutionary process and affects biodiversity patterns at all levels of biological organization. It provides populations with the potential to adapt to changes in their biotic and abiotic environment^1,2^, as confirmed in studies of experimental evolution^3^. Levels of genetic diversity have been associated to the extinction risk, and have been estimated to be 30% lower in threatened species than in their non-threatened relatives^4,5^. Moreover, genetic diversity within populations can drive ecological dynamics shaping biological communities and ecosystem functions (see^6^ for a review). For instance, some recent studies^7,8^ found significant positive effects of genetic diversity in plant populations on species richness, abundance, and productivity of associated biological communities, with important implications in applied sciences, such as ecological restoration^9^.

Given the importance of genetic diversity within populations, the analysis and interpretation of spatial patterns of variation across species ranges have been a long-lasting endeavour in evolutionary biology^10^. A major research arena has involved the identification of hotspots of intraspecific genetic diversity, that is, geographic regions harbouring exceptionally high diversity^11,12^. These hotspots are increasingly recognised as key targets in conservation biology^11, 13,14^, and their correct identification is an important step in designing effective strategies for the long-term persistence of populations in the face of global change^15^.

Despite their theoretical and applied importance, we still lack a thorough understanding of why hotspots of genetic diversity are so 'hot'. In more than thirty years of phylogeographic and population genetic investigations, hotspots of genetic diversity have often been observed in close geographic association with major Pleistocene glacial refugia. For example, southern European peninsulas, south-western and south-eastern North America, and tropical Australia have been identified as major glacial refugia as well as hotspots of biodiversity, at both species and intraspecific levels^16^. This widespread geographic association between refugia and hotspots of biodiversity has classically been viewed as evidence for a causal link between prolonged bioclimatic stability and high levels of intraspecific genetic diversity^17^. Additionally, hotspots may also result from secondary contact and admixture between intraspecific lineages, differentiated within sub-refugia during periods of unfavourable climatic conditions^17^. Under the latter scenario, hotspots of genetic diversity would in fact be melting-pots^18,19^. Although they were originally treated as alternative^11^, prolonged bioclimatic stability and secondary contact and admixture are not mutually exclusive scenarios for the formation of hotspots of intraspecific genetic diversity. In fact, substantial evidence has been gathered in favour of each scenario (e.g.^20–22^) with different levels of genetic diversity that can be explained for different populations by two factors: distance from putative refugia and extent of admixture^23,24^. However, the relative contribution of the two factors to the formation of spatial patterns of genetic variation, and particularly of hotspots, remains poorly explored (but see^11,18–22^).

Here, we address this question by analysing the contribution of bioclimatic stability and admixture following secondary contact in shaping the fine-scale spatial patterns of genetic diversity in the common toad *Bufo bufo* in peninsular Italy, a major hotspot of genetic diversity for the species^25,26^. The common toad is widely distributed in temperate habitats of the western Palearctic and range-wide phylogeographic studies have identified three divergent mtDNA lineages of the common toad in the Italian peninsula^25–27^. One lineage is restricted to southern Italy and Sicily, a second one ranges from central to north-central Italy and a third one is distributed in northern Italy and neighbouring areas. In this study we aimed to dissect the spatial patterns of genetic variation of *B. bufo* populations in its Pleistocene Italian refugium. We combined population genetic and phylogeographic tools with species distribution modelling, in order to (i) assess the fine-scale population genetic structure and diversity of *B. bufo* in the Italian peninsula; (ii) infer the location of ancestral areas, glacial refugia and secondary contact zones, and (iii) investigate the relative contribution of prolonged habitat stability and admixture between lineages in the formation and evolution of the Italian hotspot of genetic diversity for the species.

## Results

### Phylogeographic analyses based on mtDNA

**Figure 1:**
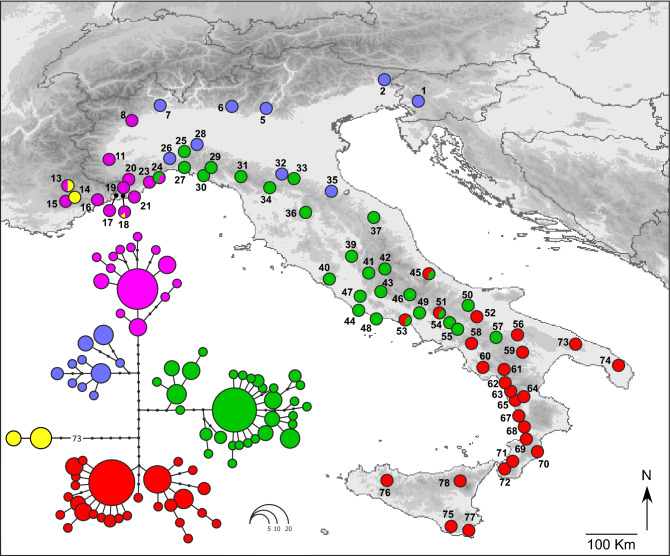
Maximum likelihood phylogenetic network of the *Bufo bufo* mitochondrial haplotypes found in Italy, and geographic distribution of the main haplotype groups. Circle sizes are proportional to haplotype frequency, and black dots represents missing intermediate haplotypes. Populations are numbered as in Table 1. The map was drawn using the software Canvas 11 (ACD Systems of America, Inc.).

We sequenced two mitochondrial DNA gene fragments in 231 *Bufo bufo* individuals from 70 sampling localities: a 722-bp fragment of the Cytochrome B gene (CytB) and a 517-bp fragment of the mitochondrial 16 s rRNA gene. In the combined mtDNA dataset (1239 bp), we found 83 different haplotypes defined by 160 variable positions. Two haplotypes found in nine individuals from three localities (13, 14 and 18) were identified as belonging to *Bufo spinosus* (Fig. 1 and Table 1).

**Table 1:** Geographic locations, sample size (n), and estimates of genetic diversity for the 78 populations of *Bufo bufo* analysed in this study. *h*: haplotype diversity; *π*: nucleotide diversity; *H*_*e*_: expected heterozygosity; *A*_*r*_; allelic richness.

Sample	Location	Latitude	Longitude	mtDNA			Microsatellites		
		(N)	(E)	n	*h*	*π*	n	*H*_*e*_	*A*_*r*_
1	Godz	45.898	13.989	5	0	0	–	–	–
2	Musi	46.316	13.251	3	0	0	10	0.443	2.932
3	Sauris	46.475	12.616	–	–	–	8	0.505	2.834
4	Monte Cesen	45.946	12.012	–	–	–	10	0.536	3.505
5	Monte Baldo	45.678	10.780	4	0.833	0.0032	7	0.524	3.202
6	Endine Gaiano	45.792	10.009	1	–	–	10	0.477	3.378
7	Arona	45.734	8.552	1	–	–	9	0.546	3.719
8	Borgofranco di Ivrea	45.511	7.873	1	–	–	–	–	–
9	Fiano (Torino)	45.219	7.540	–	–	–	10	0.469	2.986
10	Pigna	44.626	7.456	–	–	–	9	0.552	4.056
11	Barge	44.716	7.326	1	–	–	–	–	–
12	Cuneo	44.351	7.534	–	–	–	1	–	–
13	Thorame Haute	44.089	6.534	4	0.833	0.0457	7	–	–
14	Saint–Auban	43.843	6.727	5	0.600	0.0005	7	–	–
15	La Martre	43.797	6.598	5	0.400	0.0003	7	0.446	2.561
16	Gattières	43.759	7.174	5	0.600	0.0005	7	0.390	2.512
17	Rocchetta Nervina	43.883	7.603	5	0.700	0.0008	10	0.457	2.970
18	Ceriana	43.880	7.773	9	0.556	0.0273	10	0.463	2.870
19	Molini di Triora	43.988	7.776	3	1	0.0022	5	0.508	3.242
20	Mendatica	44.072	7.811	3	0	0	3	–	–
21	Lecchiore	43.916	7.921	6	0.333	0.0003	–	–	–
22	Villanova D'albegna	44.041	8.119	–	–	–	9	0.491	3.233
23	Calice Ligure	44.203	8.287	3	0.667	0.0016	10	0.557	3.371
24	Albisola Superiore	44.343	8.496	5	0.900	0.0078	10	0.413	2.515
25	Brignano-Frascata	44.829	9.038	1	–	–	–	–	–
26	Lerma	44.620	8.712	4	0.500	0.0008	10	0.572	3.777
27	San Giorgio a Bavari	44.428	9.011	1	–	–	9	0.458	3.143
28	Zavattarello	44.891	9.264	1	–	–	8	0.581	3.915
29	Varese Ligure	44.480	9.607	3	0	0	10	0.411	2.965
30	Sarzana Ligure	44.270	9.458	1	–	–	–	–	–
31	Cerreto Laghi	44.303	10.244	2	–	–	10	0.48	3.331
32	Monte San Pietro	44.360	11.108	1	–	–	10	0.569	3.994
33	Monghidoro	44.248	11.346	2	–	–	–	–	–
34	Campo Tizzoro	44.039	10.862	3	0.667	0.0016	–	–	–
35	Ciola	43.983	12.130	1	–	–	10	0.507	3.622
36	Terranuova Bracciolini	43.555	11.569	6	1	0.0034	10	0.545	3.510
37	Serra San Quirico	43.428	13.044	1	–	–	9	0.49	3.518
38	Teramo	42.687	13.721	–	–	–	10	0.476	3.086
39	San Gemini	42.608	12.558	5	1	0.0023	10	0.476	3.288
40	Canale Monterano	42.140	12.097	6	0.733	0.0016	–	–	–
41	Rocca Sinibalda	42.275	12.926	3	0	0	–	–	–
42	Scoppito	42.361	13.265	1	–	–	10	0.556	3.860
43	Jenne	41.890	13.171	5	0.900	0.0023	–	–	–
44	Bosco del Foglino	41.471	12.719	3	1	0.0011	–	–	–
45	Fara Filorum Petri	42.248	14.188	3	1	0.0081	10	0.495	3.489
46	Opi	41.791	13.807	3	1	0.0027	9	0.464	3.156
47	Doganella	41.750	12.761	4	0	0	9	0.580	3.854
48	Molella	41.268	13.046	4	0.500	0.0016	–	–	–
49	San Pietro Infine	41.444	13.968	3	0.667	0.0016	–	–	–
50	San marco la Catola	41.541	15.040	3	0	0	8	0.369	2.242
51	Lago Matese	41.409	14.405	5	1	0.0086	10	0.606	4.090
52	Biccari	41.370	15.172	3	0.667	0.0011	10	0.529	3.345
53	Grata	41.276	13.710	3	0.667	0.0075	10	0.488	3.319
54	Camposauro	41.174	14.582	5	0.800	0.0013	–	–	–
55	Tufara	41.061	14.714	3	0.667	0.0011	9	0.55	3.496
56	Spinazzola	40.998	16.059	2	–	–	7	0.573	3.951
57	Monticchio	40.929	15.603	5	0	0	10	0.485	2.386
58	Lago Laceno	40.806	15.095	8	0.607	0.0005	8	0.499	3.223
59	Tricarico	40.618	16.145	3	0.667	0.0005	9	0.499	3.158
60	Campora	40.289	15.327	3	0	0	10	0.541	3.625
61	Lago Cessuta	40.254	15.784	5	0.400	0.0003	9	0.537	3.865
62	Contrada Massadita	39.942	15.806	4	0.833	0.0008	–	–	–
63	Orsomarso	39.800	15.908	3	0	0	10	0.43	2.627
64	Lago Farneto	39.664	16.157	5	0.400	0.0003	10	0.354	2.789
65	Fagnano Castello	39.556	16.021	2	–	–	8	0.476	3.232
66	Macchialonga	39.339	16.584	–	–	–	9	0.431	2.793
67	Fiumefreddo Bruzio	39.225	16.072	5	0	0	8	0.412	3.155
68	Falerna	39.002	16.174	1	–	–	–	–	–
69	Lago dell'Angitola	38.740	16.236	2	–	–	10	0.466	2.772
70	Stilo	38.478	16.469	1	–	–	7	–	–
71	Oppido Mamertino	38.291	15.989	5	0	0	–	–	–
72	Gambarie	38.181	15.846	2	–	–	10	0.528	3.715
73	Alberobello	40.780	17.254	2	–	–	–	–	–
74	Lecce	40.345	18.167	5	0.400	0.0003	8	0.422	2.613
75	Fiume Irminio	36.929	14.674	4	0.667	0.0016	7	0.398	2.840
76	Corleone	37.869	13.305	4	0.833	0.0012	10	0.411	2.897
77	Rosolini	36.823	15.032	1	–	–	–	–	–
78	Maletto	37.853	14.831	1	–	–	–	–	–
				231			500		

The phylogenetic network outlines four main *B. bufo* haplogroups, with a clear geographic structure (Fig. 1): a north-eastern haplogroup, spanning from the eastern and central Alps to the northern side of the northern Apennines, a north-western haplogroup, restricted to the western Alps and the Provence, a central haplogroup, spanning from the northern to the central portion of the Apennine chain and a southern haplogroup, spanning from the central Apennines to the southernmost peninsular populations, including those in Sicily. The southern and central haplogroups co-occurred in the geographically intermediate localities 45, 51 and 53, whereas the north-western and central haplogroups co-occurred in a single locality (24). Haplotypes of *B. spinosus* and *B. bufo* were found co-occurring in the Provence (locality 13) and in the Ligurian Alps (locality 18).

**Figure 2:**
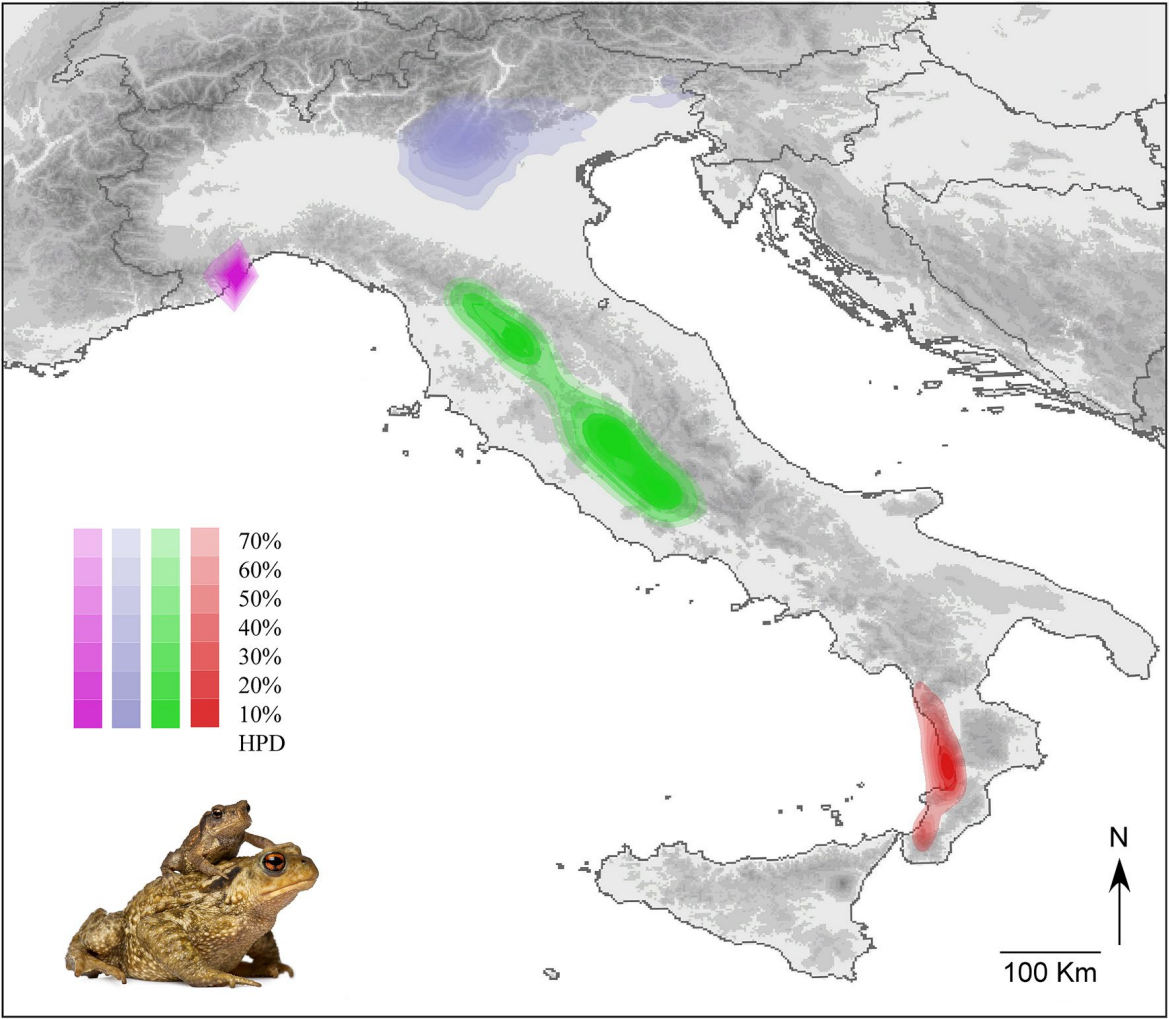
Ancestral areas of the genetic lineages of *Bufo bufo* at their respective time to the most recent common ancestor (TMRCA), as estimated by the Bayesian phylogeographical analyses. Polygons represent 10% to 70% highest posterior density (HPD) regions of the geographical locations of the ancestral areas. The map was drawn using the software Canvas 11 (ACD Systems of America, Inc.); photo: *Bufo bufo* (from https://www.dreamstime.com).

Bayesian phylogeographic analyses were conducted separately for the four main *B. bufo* mtDNA lineages. For each lineage, runs converged to a stationary distribution and had satisfactory Effective Sample Size (ESS) values (> 200). The ancestral areas of the four lineages were mapped in distant regions along the Italian peninsula (Fig. 2). The ancestral areas of the north-eastern and the north-western lineages were most likely positioned within the Ligurian Alps (time to the most recent common ancestor—TMRCA—median estimate: 418 ky, 95% HPD: 157–845 ky), and close to the Venetian Prealps (TMRCA: 416 ky, 95%HPD: 190–775 ky), respectively. The central lineage had its ancestral area projected within lowlands close to the Apennines (TMRCA: 511 ky, 95%HPD: 255–872 ky), whereas the ancestral area of the southern lineage likely occurred along the mountain massifs in the Calabria region (TMRCA: 498 ky, 95%HPD: 249–859 ky). Spatial diffusion processes from the ancestral areas likely occurred earlier for the southern and central lineages than for the two northern lineages (see Supplementary File S1). However, all the inferred events of range expansion and secondary contact among lineages occurred well before the last glacial maximum (LGM). Based on the temporal patterns of spatial diffusion, the secondary contact between the southern and the central lineages also pre-dates the last interglacial (120–140 ky), whereas the secondary contact between the central and the north-eastern lineage likely occurred close to the last interglacial.

### Population structure and genetic diversity based on microsatellite data

A total of 500 individuals from 57 populations was genotyped at nine microsatellite loci, with 3.8% of missing data. Locus *Bspi 3.02* was removed from the dataset because it tested positive for null alleles in fourteen populations. There were no significant deviations from Hardy–Weinberg or linkage equilibria after applying the Bonferroni correction for multiple tests. Across all populations, the number of alleles per locus ranged from four (*Bspi 3.11*) to 54 (*Bspi 4.29*). Allelic richness and mean expected heterozygosity estimates for each population are presented in Table 1. Population 51 (Central Italy) showed the highest values of genetic diversity, whereas the lowest values of heterozygosity and allelic richness were observed in several samples from the southernmost section of the Italian peninsula and Sicily.

**Figure 3:**
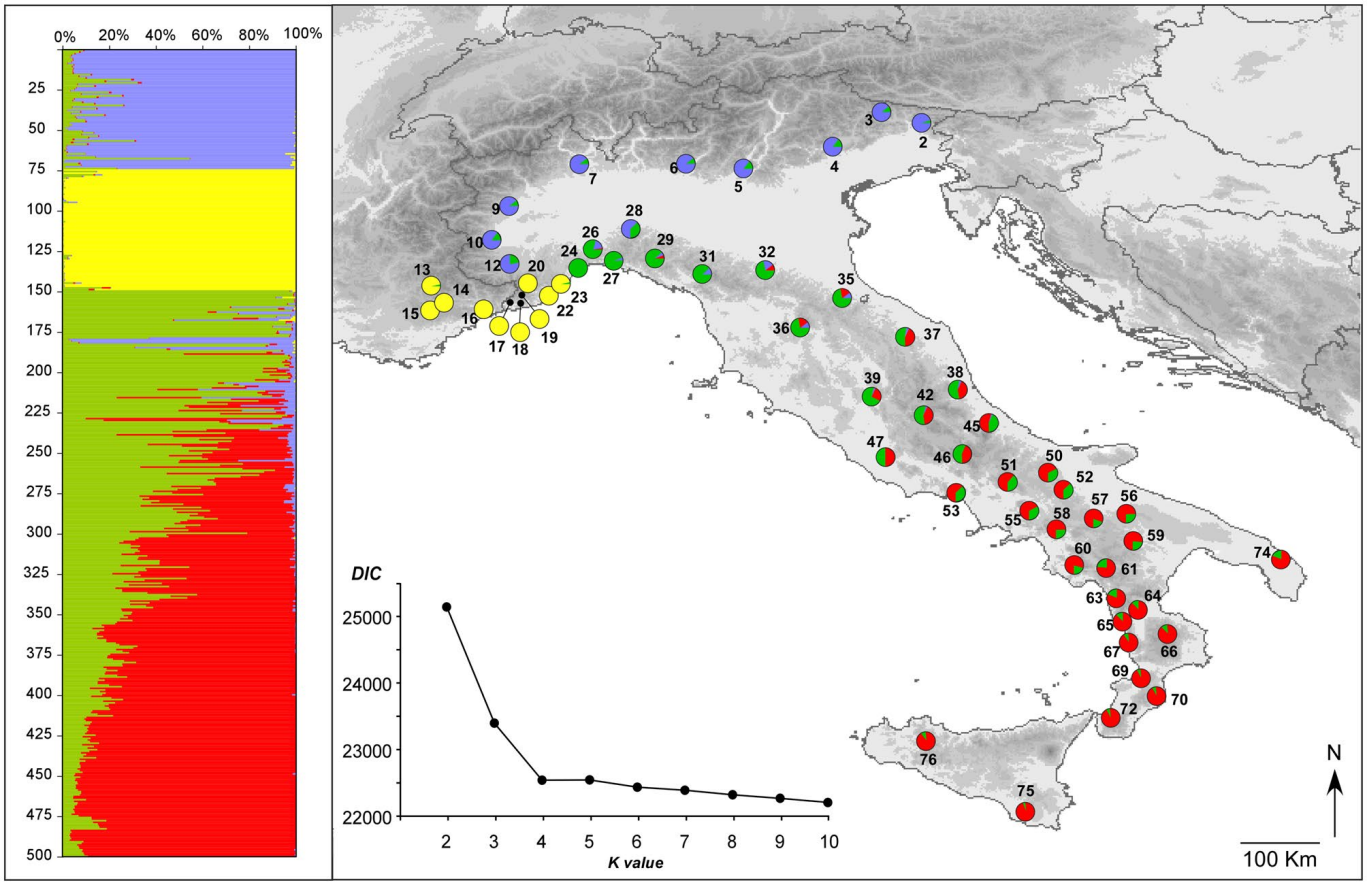
Genetic structure of the Italian *Bufo bufo* populations, as inferred by the Bayesian clustering analysis implemented in TESS based on eight microsatellite loci. The bar plot on the left shows the admixture proportions of each individual for the four genetic clusters identified; the pie diagrams on the maps show the frequency of each cluster within the studied populations. Populations are numbered as in Table 1. The line chart shows values of the deviance information criterion (DIC) statistics estimated for models with the number of genetic clusters (K) ranging from 2 to 10. The map was drawn using the software Canvas 11 (ACD Systems of America, Inc.).

TESS analyses revealed strong genetic structure in Italian *B. bufo* for the nuclear markers. The plots of values for the deviance information criterion (DIC) versus the number of clusters (K) reached a plateau at K = 4, indicating that four main genetic clusters occur in the study area (Fig. 3). The spatial distribution of these clusters shows a strong geographical signal: one cluster is widespread from the Alps to the northern side of the northern Apennines; a second cluster spans from the northern to the central Apennines; a third one spans from the central to the southern Apennines and Sicily and a fourth cluster is restricted to the Provence and to the westernmost Ligurian populations. This cluster is strongly differentiated from the other clusters and represents populations of *B. spinosus* (see also^27,28^). Bar-plots showing individual admixture proportions and pie-charts showing the average proportion of each cluster in each sampled population are presented in Fig. 3. We found widespread admixture between the southern and central genetic clusters, encompassing most of the Apennine chain. Evidence for admixture was also observed in northern populations, between the central and northern clusters. In contrast, evidence for hybridisation and admixture between *B. bufo* and *B. spinosus* was geographically restricted to their area of close proximity in the Ligurian Alps.

### Identification of glacial refugia

We obtained 581 species occurrences for the central lineage and 256 for the southern lineage (Supplementary Table S2), reduced by the thinning procedure to 369 and 154 occurrences, respectively. A total of 6655 background points for the central lineage and 4715 for the southern lineage were used in the calibration procedure.

The variance inflation factor (VIF) analysis selected seven variables for the central lineage and six variables for the southern lineage (see Supplementary Table S3). All models showed a good performance: mean AUC was 0.79 (SD = 0.02) for the central lineage and 0.81 (SD = 0.05) for the southern lineage. For both lineages, GBM was the model with the highest AUC values and GLM the one with the lowest. Model performance indices are given in Supplementary Table S3.

**Figure 4:**
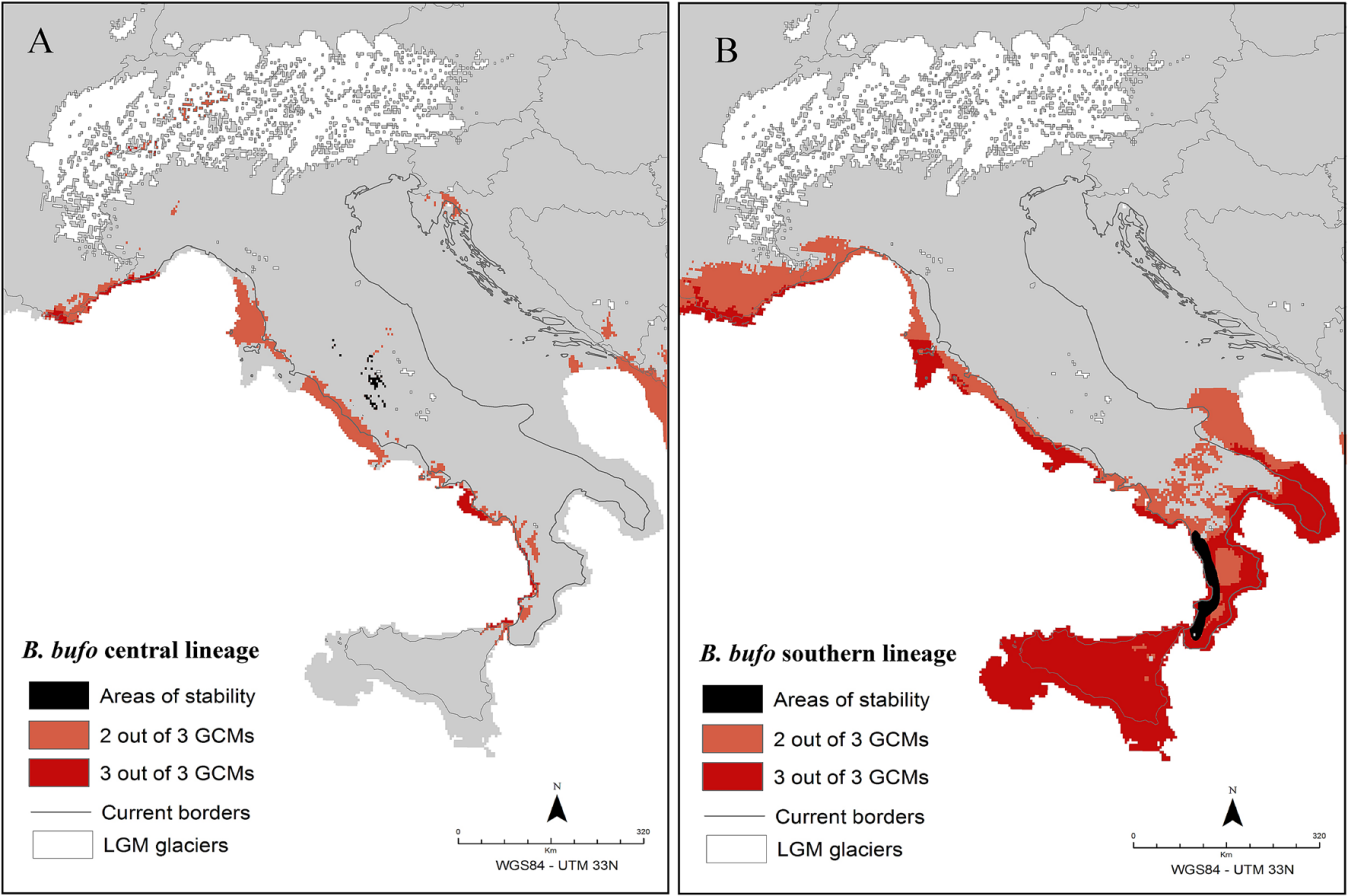
Putative Pleistocene glacial refugia for the central (**A**) and southern (**B**) lineages of *Bufo bufo*, as inferred by the species distribution modelling (SDM) calibrated under current bioclimatic conditions and projected at the last glacial maximum (21 kya). Orange: areas of at least two out of three general circulation models (GCMs); red: areas of concordance among all the GCMs; black: areas of “stability”, defined as regions of overlap between current, glacial (two out of three GCMs) and ancestral (Bayesian phylogeographic analysis) areas of species presence. The map was drawn using the software Canvas 11 (ACD Systems of America, Inc.).

The current SDM is consistent with previous knowledge about the distribution of the common toad^29^: high suitability areas are scattered throughout most of the Italian peninsula, where the species is more common at medium elevations and rare and isolated in the main plains and at higher elevations (see Supplementary Figure S1). The putative climatic refugia for the southern lineage at the LGM (Fig. 4 and Supplementary Figure S2) covered most of the Calabrian and Apulian coastlines (predicted by all GCMs), as well as the inland of the same areas (predicted by two GCMs). The putative glacial refugia for the central lineage only covered a small area along the Tyrrhenian coastline in Tuscany (predicted by two GCMs).

### Predictors of genetic diversity

Results from the generalised linear models (GLMs) are summarised in Table 2. Irrespective of the genetic diversity index used as dependent variable, admixture between divergent lineages was the only significant predictor of population genetic diversity. On the contrary, refugia and areas of stability showed no significant effects on genetic diversity.

**Table 2:** Outcomes of the generalized linear mixed models.

GLM	Coefficients			ANOVA			
	Variable	Estimate	Pr( >|t|)	Df	Df Resid	F	Pr(> F)
***A***_***r***_** ~ stability + refugia + Simpson**							
AIC = 49.395	stability	− 3.44E−04	0.706	1	34	0.380	0.542
	refugia	1.14E−03	0.698	1	33	32.017	0.083
	Simpson	1.31E + 03	**0.025**	1	32	55.691	**0.025**
***A***_***r***_** ~ stability + Simpson**							
AIC = 47.567	stability	− 1.63E−04	0.833	1	34	0.390	0.537
	Simpson	1.42E + 03	**0.005**	1	33	88.447	**0.005**
***A***_***r***_** ~ refugia + Simpson**							
AIC = 47.557	refugia	5.74E−04	0.818	1	34	19.373	0.173
	Simpson	1.38E + 03	**0.011**	1	33	73.081	**0.011**
***A***_***r***_** ~ stability**							
AIC = 54.115	stability	− 4.75E−04	0.577	1	34	0.317	0.577
***A***_***r***_** ~ refugia**							
AIC = 52.759	refugia	3.17E−03	0.210	1	34	16.342	0.210
***A***_***r***_** ~ Simpson**							
AIC = 45.616	Simpson	14.292	**0.004**	1	34	94.549	**0.004**
***H***_***e***_** ~ stability + refugia + Simpson**							
AIC = − 99.234	stability	4.24E−05	0.715	1	34	0.086	0.772
	refugia	− 1.36E−04	0.716	1	33	22.243	0.146
	Simpson	2.37E + 02	**0.002**	1	32	113.730	**0.002**
***H***_***e***_** ~ stability + Simpson**							
AIC = − 101.08	stability	2.08E−05	0.832	1	34	0.088	0.769
	Simpson	2.25E + 02	**0.001**	1	33	138.244	**0.001**
***H***_***e***_** ~ refugia + Simpson**							
AIC = − 101.08	refugia	− 6.62E−05	0.834	1	34	15.699	0.219
	Simpson	2.28E + 02	**0.001**	1	33	123.410	**0.001**
***H***_***e***_** ~ stability**							
AIC = − 90.486	stability	− 2.86E−05	0.802	1	34	0.064	0.802
***H***_***e***_** ~ refugia**							
AIC = − 91.644	refugia	3.63E−04	0.286	1	34	11.772	0.286
***H***_***e***_** ~ Simpson**							
AIC = − 103.033	Simpson	0.22274	**0.001**	1	34	14.267	**0.001**

Allelic richness (*A*_*r*_) and expected heterozygosity (*H*_*e*_) were entered as dependent variables; distance from the nearest glacial refugium, distance from the nearest stability area, Simpson’s diversity index, and their interactions were entered as predictors.

Significant factors are shown in bold.

AIC, Akaike Information Criterion.

## Discussion

In line with previous studies^25,26,28^, mitochondrial and microsatellite markers consistently identified three main lineages of *Bufo bufo*, ranging in southern, central and northern Italy. The phylogeographic boundaries between lineages, although sharper with mtDNA than with the microsatellite markers, coincide with two well-known suture zones of the Italian peninsula: the lower Volturno-Calore river basin and the northern Apennines^12^. The extent of phylogeographic concordance with previously studied organisms from the same area is substantial, in terms of the co-distribution of intraspecific lineages^20,30–37^. However, the spatial pattern of genetic variation observed within *B. bufo* populations shows some remarkable features, that may well exemplify the disparity between co-distribution of phylogeographic lineages and correlated population histories.

The geographic distribution of the southern lineage is bounded by the island of Sicily to the south and the lower Volturno-Calore river basin in central Italy to the north. This distribution closely matches phylogeographic patterns in co-distributed taxa (e.g.^20,30–34^). However, the spatial distribution of genetic variation within the range of the southern lineage of *B. bufo* contrasts with that found in other species studied with enough sampling depth, typically comprising finer population structure and multiple population units arranged along the south-north axis^18,20,31,34–38^. The south of the Italian peninsula has been recurrently identified as a hotspot of intraspecific genetic diversity^14,18,31^. Conversely, neither mtDNA nor microsatellites provided evidence for further population structure within the southern lineage of *B. bufo*, and, accordingly, the southernmost area of the peninsula is rather a ‘cold spot’ of genetic diversity for this species (Table 1). Throughout the Plio-Pleistocene, the southern portion of the Italian peninsula was repeatedly fragmented by glacio-eustatic marine transgressions^39^. These cycles of fragmentation into paleo-islands followed by their re-assembly, have had a major impact on the evolution of the regional temperate biota, likely including *B. bufo* populations. The lower Volturno-Calore river basin was precisely the northernmost site affected by repeated marine transgressions, which plausibly played a role in the divergence between the southern and the central lineages of *B. bufo* (estimated at 1.7 My ago^26^). The absence of population structure of the extant *B. bufo* lineage in southern Italy may result from two processes. Either the range expansion of the extant southern lineage swamped all other pre-existing population units, or these went extinct before this range expansion (see Supplementary Figure S3 and Supplementary File S1). In the former case, however, admixture among divergent population units during the expansion should have inflated genetic diversity in southern Italy, at least in the nuclear genome, as observed in other taxa^18,31^. Thus, the minimal level of genetic diversity observed in this area fits better with a scenario of early disappearance of—probably small and insular—population units prior to the most recent expansion event in southern Italy and Sicily.

The geographic distribution of the central lineage parallels the distribution of intraspecific lineages in other organisms, including reptiles^34^ and amphibians^26–37^, as well as several cryptic endemic species^32^. In this case, however, concordance also involves the spatial and temporal features of phylogeographic reconstructions. For example, *Triturus carnifex*^35^ and *Hyla intermedia*^36^ have intraspecific lineages (i) co-distributed with the central lineage of *B. bufo*, (ii) with closest affinities with lineages in southern Italy, and (iii) not showing evidence of further population sub-structure within this area. Whether these multiple lines of phylogeographic concordance are genuine realisations of correlated population histories in response to paleoenvironmental changes requires further research.

At first glance, the geographic distribution of the northern lineage, albeit incompletely captured by our sampling design (see^27^), appears in concordance with the distribution of several phylogeographic lineages sandwiched between the Alps and the northern Apennines^34–36,44^. However, in *B. bufo* this probably involved a unique biogeographic route, encompassing two distinct colonisation events of the river Po plain, one from the east and one from the west. Indeed, this lineage is characterised by a single nuclear gene pool (see also^28^), but two distinct mtDNA sub-lineages, with western and central—eastern European distributions, respectively, coincident with the *e2* and *e3* lineages of^25^. Arntzen et al.^27^ suggested that the two colonisation events occurred after the LGM, eastward from a western refugium, and westward from the northern Balkans. Our results support this scenario, with some amendment. We modelled two distinct ancestral areas for the two mtDNA sub-clades, and we located one area in the Ligurian Alps, and one area close to the Venetian Prealps, respectively (Fig. 2). However, our Bayesian phylogeographic reconstructions (Supplementary File S1) showed that the eastern sub-clade completed most of its range expansion before the last glacial cycle, whereas the western sub-clade completed most of its range expansion during the last glaciation. Finally, the absence of two sub-groups of *B. bufo* in northern Italy in the nuclear dataset, and the occurrence of *B. spinosus* in the putative range of the *e2* sub-clade (Fig. 3) might be explained by a late expansion of *B. spinosus* in the north-west, which then hybridised with local *B. bufo* populations picking up the *e2* mtDNA lineage in the process^27,28^.

Our correlative models indicate that the distance from glacial refugia or stability areas do not contribute much to explaining levels of genetic diversity within *B. bufo* populations in peninsular Italy, irrespective of the genetic diversity index analysed. These results do not deny a role for glacial refugia in preserving the spatial pattern of genetic diversity, but emphasize the importance of other factors, like admixture between well-differentiated lineages.

The GLMs (Table 2) indicate that the differences between populations in terms of levels of genetic diversity are best explained by the extent of admixture between distinct lineages occurring within each studied population. A key role for secondary contact and admixture in shaping spatial patterns of population genetic diversity, although not formerly analysed quantitatively in terms of effect size, has emerged in several previous studies for species from the Italian peninsula (see references above), as well as from the other Mediterranean peninsulas of Iberia and the Balkans^12,23,24^. In most of these studies, however, the secondary contact phase was estimated to have occurred in the late-glacial or post-glacial epochs, or its timeline was not estimated at all. Our Bayesian phylogeographic reconstruction clearly indicated that the range expansion events setting the stage for secondary contacts and gene exchanges initiated, and were most probably completed, well before the last glacial maximum. In the northern Apennine, the co-occurrence of distinct lineages was probably established early in the last interglacial phase, while in the lower Volturno-Calore river basin it most probably occurred before, during the late Middle Pleistocene. This historical scenario has at least one major albeit non-intuitive implication, that is, the spatial patterns of intergradation between lineages and of genetic diversity within populations observed in peninsular Italy is of ancient origin. Therefore, these patterns would have survived at least one glacial-interglacial cycle (probably more) and the substantial range variations that, according to our SDM analyses, affected the *B. bufo* lineages in peninsular Italy in the Late Pleistocene. As mentioned above, there is an increasing appreciation of the major role and long-term consequences of secondary contact and admixture processes in increasing the genetic diversity of populations, promoting the formation of hotspots, as well as the sharing of adaptive genetic material between divergent lineages. However, in the absence of barriers to gene flow between the interacting lineages, the spatial patterns associated to these historical processes are mostly seen as transient^45,46^. In fact, the scenario emerging from our results suggests that they might be not so ephemeral as usually thought.

According to the estimated SDMs for the central and southern lineages at the LGM (Fig. 4), a large area of high bioclimatic suitability for the species was located in southern Italy. However, a coastal strip of suitable bioclimatic conditions also occurred along the western side of the peninsula. A comparison of the SDMs estimated for the central and southern lineages suggests that this coastal strip might have run seamless from south to north, preventing a cyclical fragmentation of the *B. bufo* populations into separate glacial refugia along the south-north axis. Notably, much of this coastal strip is located within areas that are presently below the sea-level, indicating that it acted as a "true refugium" sensu Recuero & García-París^47^, that is, as an area previously unoccupied by the species, where lineages retreat once their range becomes unsuitable due to paleoclimatic changes.

Together with the very limited and highly fragmented distribution of the inferred areas of stability (see Fig. 4), this scenario might also explain why our correlative models found no evidence of a minimal role for glacial refugia and climatically stable areas in explaining the observed pattern of population genetic diversity. Indeed, we didn’t find evidence of a real “sanctuary-type” refugium, that is, of a large area (or multiple areas) of persistently suitable bioclimatic conditions, where populations persisted over multiple episodes of climatic change^47^. Instead, throughout most of the peninsula, *B. bufo* populations likely moved from interior areas (and probably higher altitudes) toward coastal regions and back, in response to paleoclimatic oscillations, through small-scale range migrations that did not erase previously formed patterns of genetic variation. Under this scenario, glacial refugia contributed to preserving levels and patterns of genetic diversity across glacial-interglacial cycles, but not to their formation.

Finally, our results extend previous findings concerning the possible location of glacial refugia within areas that are currently covered by the sea. Indeed, although the occurrence of such areas has already been postulated, they have been mostly linked to insular geographic settings^48–50^, or to continental areas where wide coastal lowlands opened following glacial sea-level drops^35,43,51,52^. In line with these previous studies, our SDMs for the LGM suggested a possible area of suitable bioclimatic conditions for the southern lineage at the southern edge of the wide coastal lowland now covered by the Adriatic Sea. However, as discussed above, we also found evidence of a narrow glacial refugium along the Tyrrhenian (glacial) coast, which is at the same time an unprecedented but not completely unexpected result. Indeed, paleoenvironmental reconstructions based on palynological, microfossils, and sedimentological data, show the existence of areas of high ecological stability along the western coastal plains of the peninsula^53,54^, where the effects of climate change were mitigated^54^. Our results suggest that the importance of these narrow coastal refugia for temperate animal species might have been overlooked in previous phylogeographic studies of co-distributed species^35,36,51^.

## Conclusions

Why are hotpots of genetic diversity so hot? From the perspective of the common toad *B. bufo* in peninsular Italy the answer is they are because they have been melting pots. Contrary to our expectations, based on previous studies of co-distributed species, we did not find a hotspot of genetic diversity in the southernmost area of the Italian peninsula, a well-documented glacial refugium for a broad range of taxa. Instead, populations from this area were among the least variable. Overall, spatial patterns of population genetic diversity were linked with the extent of admixture between distinct intraspecific lineages rather than with climatically stable areas. This highlights a general principle emerging from the documentation of concordant phylogeographic patterns in species with disparate evolutionary histories in southern European peninsulas: higher levels of genetic diversity mark populations with substantial genetic contributions from multiple differentiated lineages, irrespective of their location regarding glacial refugia.

## Materials and methods

### Sampling and laboratory procedures

We collected 563 *Bufo bufo* individuals from 78 sampling localities (Table 1 and Fig. 1). Tissue samples were collected as tail- or toe-clips from tadpoles or adult individuals respectively, which were then released in the respective collection sites. Samples were stored in 95% ethanol until DNA extraction. Field works, collection of tissues, and the experimental protocols were approved by the Italian Ministry of Environment (Permit Numbers: DPN-2009-0026530) and were performed in accordance with the relevant guidelines and regulations (including ethics guidelines and regulations).

DNA extractions were carried out using commercial kits (ZYMO RESEARCH). We amplified by polymerase chain reaction (PCR) two mitochondrial DNA (mtDNA) fragments, a 722-bp fragment of the Cytochrome B gene (*CytB*) and a 517-bp fragment of the mitochondrial 16 s rRNA gene. Amplifications were performed in a 15-μL reaction volume following protocols described in Recuero et al.^26^. Purification and sequencing of the PCR products were conducted by Macrogen Inc. (http://www.macrogen.com), using an ABI PRISM 3730 sequencing system (Applied Biosystems). Electropherograms were checked by eye using FinchTV 1.4.0 (Geospiza Inc.). All sequences were deposited in the GenBank database (Supplementary Table S1).

Patterns of genetic variation in the nuclear genome were investigated with nine microsatellite loci (*Bspi 3.02*, *Bspi 3.26*, *Bspi 4.30*, *Bspi 3.19*, *Bspi 4.16*, *Bspi 3.11*, *Bspi 4.27*, *Bspi 4.14* and *Bspi 4.29*), following previously described protocols^55^. Other markers known from the literature (see^55^, and references therein) were excluded from the analysis after trials based on 96 individuals showed inconsistent amplification in > 30% of the individuals analysed. Forward primers were fluorescently labelled and PCR products were electrophoresed by Macrogen Inc. on an ABI 3730xl genetic analyser (Applied Biosystems) with a 400-HD-size standard.

### Phylogeographic analyses based on mtDNA

MtDNA sequences were aligned using GeneStudio Pro 2.2.0.0 (GeneStudio Inc., Suwanee, GA). Phylogenetic relationships between mtDNA haplotypes were inferred using the maximum likelihood (ML) method implemented in PhyML3.10^56^, applying the Neighbour Nearest Interchange method for tree improvement and the best substitution model (TrN93 + G) selected by the Smart Model Selection procedure^57^ under the Bayesian Information Criterion. The robustness of the topology was assessed via 1000 bootstrap pseudo-replicates. The estimated tree topology was then converted into a haplotype genealogy using Haplotype Viewer^58^.

The ancestral areas of the main *B. bufo* genetic lineages and the spatial and temporal patterns of diffusion throughout their range were estimated using the Bayesian phylogeographic (BP) analysis in continuous space implemented in BEAST 1.8^59^. To avoid any potential bias caused by population structure^60^, we performed separate analyses with the same settings for each main haplogroup identified by the previous phylogenetic analysis. We set the Bayesian skyline as coalescent tree prior^61^, MCMCs with length of 200 million generations sampling every 20 000 generations, “Cauchy” as spatial diffusion model^59,62^, a strict molecular clock model, and the fossil-calibrated substitution rate of 5.5 × 10^–9^ substitutions/year^26^. Geographical coordinates were provided for each individual, applying a jitter of ± 0.001° to duplicated coordinates. Trace files were inspected using Tracer 1.6^63^ to evaluate the Effective Sample Size (ESS) of the estimated parameters, the appropriate burn-in, and the convergence between runs. Finally, the full posterior sample of trees was analysed in SPREAD 1.0.7^64^, in order to estimate the ancestral area for each lineage (i.e. the geographic location of its most recent common ancestor).

### Population structure and genetic diversity based on microsatellite data

Microsatellite data were analysed using GENEMAPPER 4.1. Micro-Checker 2.2.3^65^ was used to test for the presence of null alleles and large-allele dropout. Allelic frequencies, tests for deviations from the expected Hardy–Weinberg and linkage equilibria, and estimates of allelic richness (Ar; computed using the rarefaction method) and the mean observed and expected heterozygosity (Ho and He) were computed using the *diveRsity* R package^66^, after excluding populations of sample size n < 4 in at least one locus (localities 12, 13, 14, 20, and 70).

The population genetic structure was evaluated by means of the Bayesian clustering algorithm implemented in TESS 2.3.1, with the geographical origin of individuals as prior information^67^. The analysis was carried out by modelling admixture using a conditional autoregressive model, and consisted of 100 replicates for each K value (i.e. the number of clusters) between 2 and 10. Each replicate was 50 000 steps long, and the first 20 000 steps were discarded as burn-in. The spatial interaction parameter was kept at the default value (0.6), with the update option activated. The model that best fit the data was selected using the deviance information criterion (DIC). DIC values were averaged over 100 replicates for each value of K, and the best K value was selected as the one at which the average DIC reached a plateau. For the selected K value, the estimated admixture proportions of the 10 runs with the lowest DIC were averaged using CLUMPP 1.1.2^68^.

### Identification of glacial refugia

Locations of glacial refugia during the Last Glacial Maximum (LGM) were estimated with species distribution models (SDM) calibrated on the current climate and projected on the LGM. All SDMs were developed using the *biomod2* R package, following an ensemble forecasting approach^69^. To account for intraspecific variability^70–72^, we calibrated distinct SDMs for the central and southern lineages, because they are the only lineages whose geographic distribution has been fully resolved (see Results section). Occurrence data for the entire geographic distribution of each lineage were obtained by pooling field data collected during this study together with data from: Stoch^73^, Global Biodiversity Information Facility (GBIF.org, 06 April 2017—GBIF Occurrence Download https://doi.org/10.15468/dl.wyiqnj), Observado (www.observation.org). Occurrence data from Stoch^73^ were validated with our own field observations or by matching toponomy with topography and satellite imagery information, reaching precision ≥ 30 arcseconds (≈1 km at the equator); occurrence data from GBIF were verified by inspecting associated toad pictures. Duplicated records and data georeferenced with uncertainty ≥ 30 arcseconds (≈1 km at the equator) were removed.

To limit spatial autocorrelation among occurrences^74^, we thinned the raw occurrences using the *spThin* R package^75^, obtaining five alternative calibration datasets for each evolutionary lineage. We thinned the occurrences considering the nearest neighbour distance expected in the study area with our sampling effort for a random sample (5.97 km for the central lineage, 9.59 km for the southern lineage along the peninsula, 15.06 km for the southern lineage in Sicily). To limit the effect of sampling bias, we used a target-group approach to inform the selection of background data points^76^. We collected all data available for reptiles and amphibians available from the same references and for the same study area, obtaining a total of 12,360 unique records; these records were divided following the geographical distribution of each lineage and used in model calibration as background points.

SDMs were calibrated considering the full set of 19 bioclimatic variables (current climate) obtained at 30-arc-second resolution from the WorldClim database (http:\\www.worldclim.org77). We did not include in the analyses elevation data, which are highly collinear with temperature and would represent a problem for model transferability in time^74^, or NDVI (a satellite based index of the photosyntetically active vegetation), for which no corresponding layer exists back in time. To avoid issues linked to multicollinearity^73^, we performed a variance inflation factor (VIF) analysis, retaining only variables with VIF < 5. The same variables were also obtained for the LGM climate from the same database (at resolution 2.5′) according to three general circulation models (GCM: CCSM4, MIROC-ESM, MPI-ESMP-P).

For each lineage, we calibrated an ensemble SDM considering multiple initial conditions (the five thinned sets of occurrence points coupled with the set of background points and with the same climate layers) and different model classes (generalised linear model, GLM; generalised additive model, GAM; generalised boosted model, GBM; multivariate adaptive regression spline, MARS; maximum entropy model, MAXENT.Phillips). All models were validated using the occurrences excluded in the thinning; in particular we measured the area under the curve (AUC) of the receiver operating characteristic (ROC) and calculated the probability threshold which maximize the true skill statistics (maxTSS^78^). The final ensemble model was calculated as the AUC-weighted average of all models with AUC value greater than 0.7^79^ and then projected under the current climate over the study area.

Finally, the same models were projected over the study area also considering LGM climate, obtaining a separate ensemble model for each available GCM. Each model was transformed into a binary model using the maxTSS threshold. We defined as putative glacial refugia all pixels in the Italian peninsula classified as one by at least two GCMs.

### Predictors of the spatial patterns of genetic diversity

To explore the relative contribution of genetic admixture between divergent lineages and long-term bioclimatic stability within refugia to current levels of population genetic diversity, we used generalised linear models (GLM) carried out by means of the “glm” function in the *lme4* R package. Because we could not estimate the geographic location of glacial refugia in northern Italy (see previous section), subsequent analyses were carried out on the reduced dataset including only populations from the Apennine peninsula and Sicily (localities 24–76).

The extent of admixture within each population (i.e. sampling site) was estimated by calculating the Simpson’s diversity index^80^, based on the number of genetic clusters (i.e. ancestral lineages) and their proportional contribution to the genetic make-up of each sampled population, as previously estimated by the TESS analysis.

In order to address the contribution of long-term bioclimatic stability to levels of population genetic diversity, we estimated two Euclidean distance measures: (i) distance of each sampled population to the nearest area of glacial refugium, as estimated by the SDM analysis; (ii) distance of each population to the nearest “stability area”, defined as a region of overlap between a glacial refugium and an ancestral area (as previously inferred by the Bayesian phylogeographic analysis). The latter regions were considered as our best estimates for the areas of long-term bioclimatic stability (i.e. stability areas), since all the time windows available (i.e. current, LGM and TMRCA) support the species occurrence in these regions. However, it is worth mentioning that this procedure for the identification of stability areas might overestimate the occurrence of these areas, as in the case of repeated population extinctions and recolonizations in time-windows not covered by previous SDM and Bayesian phylogeographic analyses. Euclidean distances separating each studied population from these areas were computed using the function “*near*” in ArcGIS 10.1 (ESRI).

Two series of GLMs with Gaussian distribution and identity link function were built, one using allelic richness and one using expected heterozygosity as dependent variables. GLMs were conducted using as predictors: Simpson’s index, the distance from the nearest refugium, the distance from the nearest stability area, as well as their interactions. Model comparisons were carried out by means of the Akaike Information Criterion.

## Supplementary Information

Supplementary Figure S1.

Supplementary Figure S2.

Supplementary Figure S3.

Supplementary File S1.

Supplementary Table S1.

Supplementary Table S2.

Supplementary Table S3.

## Data Availability

DNA sequences used in the present study are deposited in GenBank. Supporting Information files are available in the online version of this article and have been deposited at the Dryad Data Repository (10.5061/dryad.qz612jmc6).
