# Publisher Correction: Reconstructing hotspots of genetic diversity from glacial refugia and subsequent dispersal in Italian common toads (*Bufo bufo*)

**DOI:** 10.1038/s41598-021-94521-w

**Published:** 2021-07-20

**Authors:** Andrea Chiocchio, Jan. W. Arntzen, Iñigo Martínez‑Solano, Wouter de Vries, Roberta Bisconti, Alice Pezzarossa, Luigi Maiorano, Daniele Canestrelli

**Affiliations:** 1 Department of Ecological and Biological Science, Tuscia University, Largo dell’Università s.n.c., 01100 Viterbo, Italy; 2 Naturalis Biodiversity Center, P.O. Box 9517, 2300 RA Leiden, The Netherlands; 3 Department of Biodiversity and Evolutionary Biology, Museo Nacional de Ciencias Naturales, CSIC, c/ José Gutiérrez Abascal 2, 28006 Madrid, Spain; 4 Asociation Ambor, Ctra. Constantina – Pedroso 1, 41450 Constantina, Spain; 5 Department of Biology and Biotechnology “Charles Darwin”, Università di Roma La Sapienza, Viale dell’Università 32, 00185 Rome, Italy

Correction to: *Scientific Reports* 10.1038/s41598-020-79046-y, published online 08 January 2021

The original version of this Article contained an error in Figure 1 and Figure 3 where parts of the figures were incorrectly captured. The original Figure 1 and Figure 3 and accompanying legends appear below.

**Figure 1:**
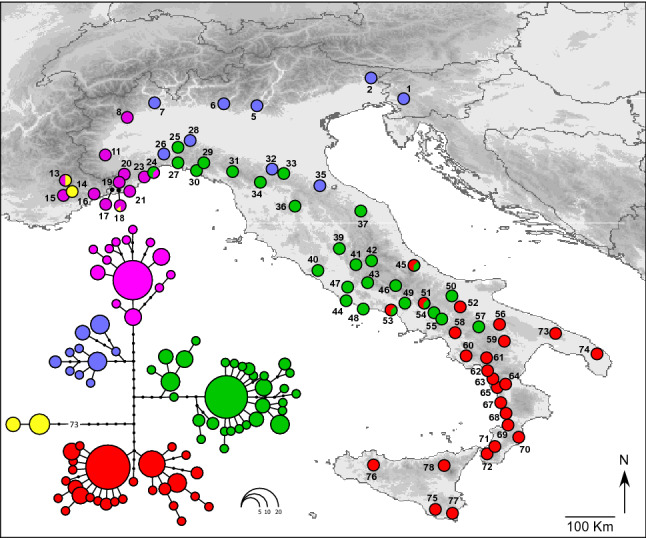
Maximum likelihood phylogenetic network of the *Bufo bufo* mitochondrial haplotypes found in Italy, and geographic distribution of the main haplotype groups. Circle sizes are proportional to haplotype frequency, and black dots represents missing intermediate haplotypes. Populations are numbered as in Table 1. The map was drawn using the software Canvas 11 (ACD Systems of America, Inc.).

**Figure 3:**
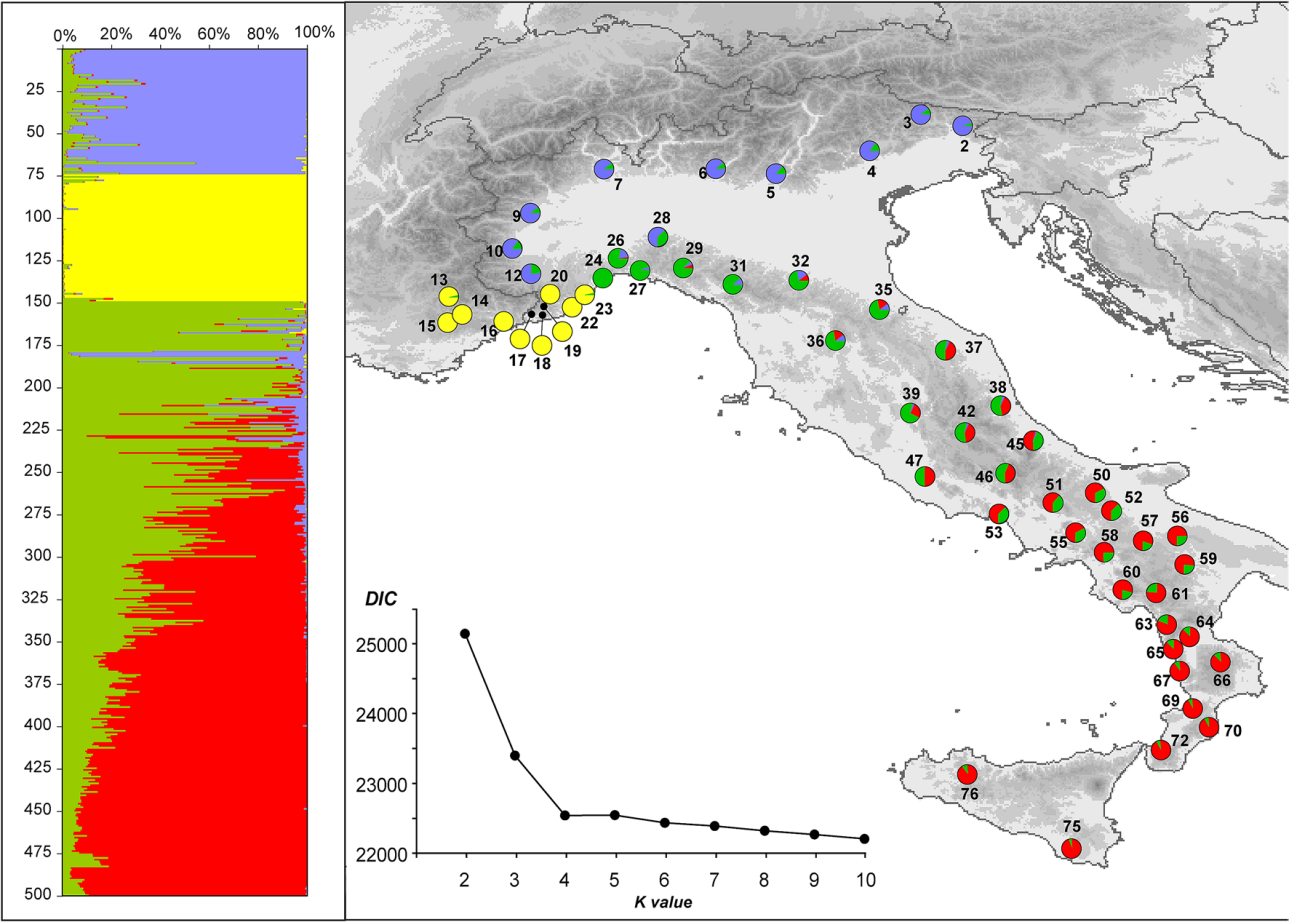
Genetic structure of the Italian *Bufo bufo* populations, as inferred by the Bayesian clustering analysis implemented in TESS based on eight microsatellite loci. The bar plot on the left shows the admixture proportions of each individual for the four genetic clusters identified; the pie diagrams on the maps show the frequency of each cluster within the studied populations. Populations are numbered as in Table 1. The line chart shows values of the deviance information criterion (DIC) statistics estimated for models with the number of genetic clusters (K) ranging from 2 to 10. The map was drawn using the software Canvas 11 (ACD Systems of America, Inc.).

The original Article has been corrected.

