# Supplementary material for: Reconstructing hotspots of genetic diversity from glacial refugia and subsequent dispersal in Italian common toads (Bufo bufo)
Source: Sci Rep. 2021 Jan 8;11:260. doi: 10.1038/s41598-020-79046-y (PMC7794404; doi:10.1038/s41598-020-79046-y)

Scientific Reports

SUPPORTING INFORMATION

Reconstructing hotspots of genetic diversity from glacial refugia and subsequent dispersal in Italian common toads (*Bufo bufo*)

Andrea Chiocchio, Jan. W. Arntzen, Iñigo Martínez-Solano, Wouter de Vries, Roberta Bisconti, Alice Pezzarossa, Luigi Maiorano, Daniele Canestrelli

Supplementary Figure S1 – SDM projection under current bioclimatic conditions

Figure S1 – Projections of the species distribution model (continuous maps) under the current bioclimatic conditions for the central lineage (left) and the southern lineage (right). Maps were generated in ArcGIS 10.1.

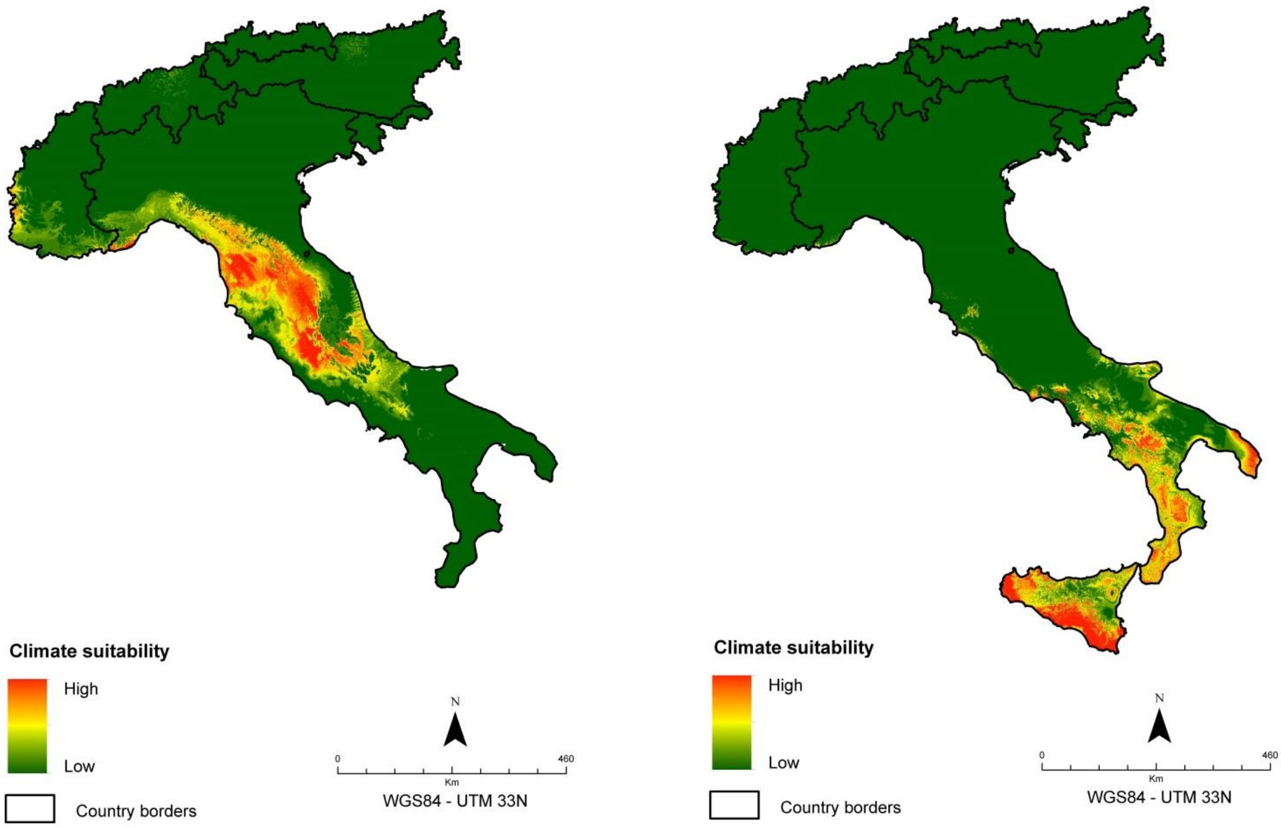

Supplement: Supplementary file 1 — Supplementary Figure S1. [file 41598_2020_79046_MOESM1_ESM.pdf]
