# Supplementary material for: Reconstructing hotspots of genetic diversity from glacial refugia and subsequent dispersal in Italian common toads (Bufo bufo)
Source: Sci Rep. 2021 Jan 8;11:260. doi: 10.1038/s41598-020-79046-y (PMC7794404; doi:10.1038/s41598-020-79046-y)
Supplement: Supplementary file 2 — Supplementary Figure S2. [file 41598_2020_79046_MOESM2_ESM.pdf]

Scientific Reports

SUPPORTING INFORMATION

Reconstructing hotspots of genetic diversity from glacial refugia and subsequent dispersal in Italian common toads (*Bufo bufo*)

Andrea Chiocchio, Jan. W. Arntzen, Iñigo Martínez-Solano, Wouter de Vries, Roberta Bisconti, Alice Pezzarossa, Luigi Maiorano, Daniele Canestrelli

Supplementary Figure S2 – SDM projection under LGM bioclimatic conditions

Supplementary Figure S2 – Projections of the species distribution model at the last glacial maximum, for the central lineage (left) and the southern lineage (right), according to the three global circulation models used in this study (CCSM4, MIROC, MPI); binary maps, maxTSS thresholds: central lineage, 0.36; southern lineage, 0.46. Maps were generated in ArcGIS 10.1.

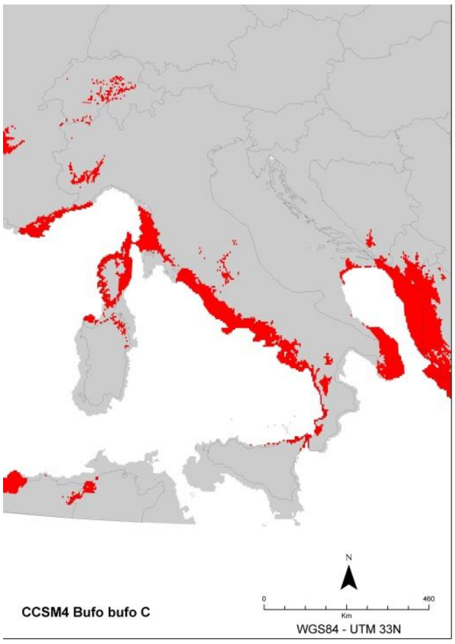

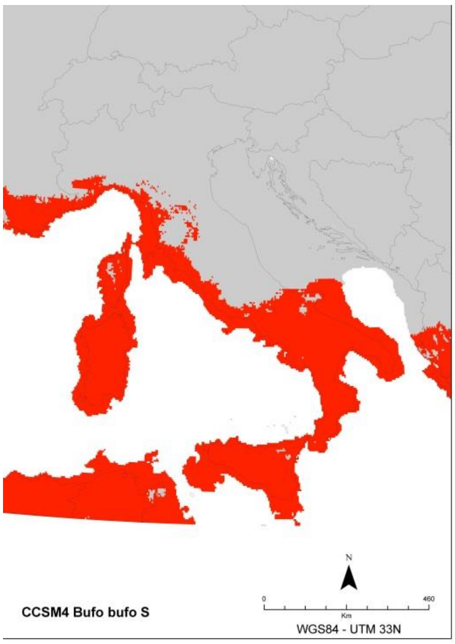

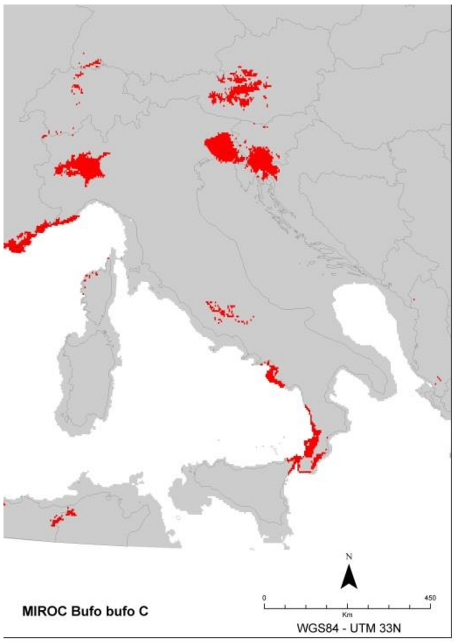

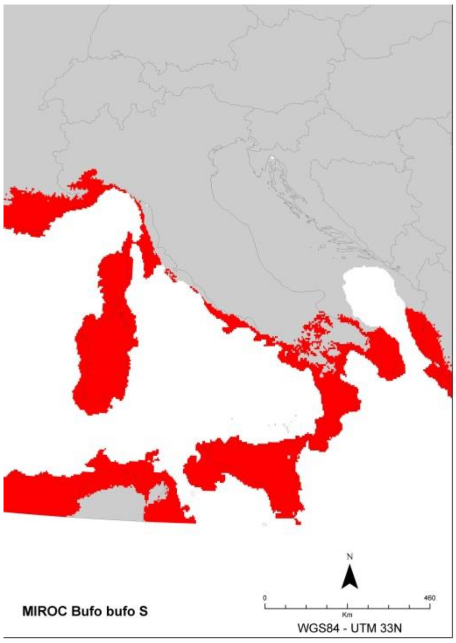

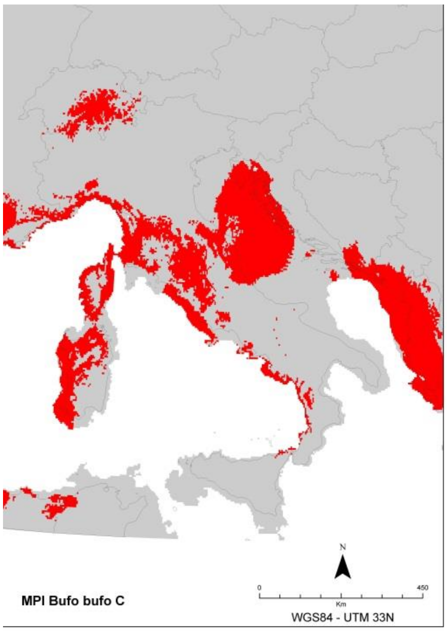
MPI Bufo bufo C
0
450
Km
WGS84 - UTM 33N
N

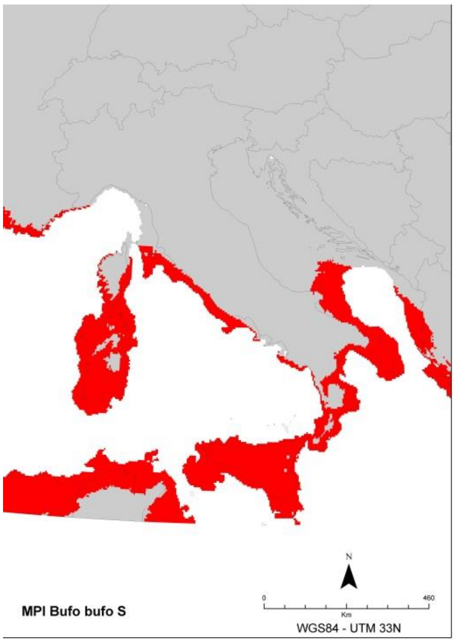
MPI Bufo bufo S
0
460
Km
WGS84 - UTM 33N
N
