# Supplementary material for: Reconstructing hotspots of genetic diversity from glacial refugia and subsequent dispersal in Italian common toads (Bufo bufo)
Source: Sci Rep. 2021 Jan 8;11:260. doi: 10.1038/s41598-020-79046-y (PMC7794404; doi:10.1038/s41598-020-79046-y)

Scientific Reports

SUPPORTING INFORMATION

Reconstructing hotspots of genetic diversity from glacial refugia and subsequent dispersal in Italian common toads (*Bufo bufo*)

Andrea Chiocchio, Jan. W. Arntzen, Iñigo Martínez-Solano, Wouter de Vries, Roberta Bisconti, Alice Pezzarossa, Luigi Maiorano, Daniele Canestrelli

Supplementary Figure S3 – Bayesian phylogeograpy reconstructions

Supplementary Figure S3 – Spatial diffusion of *Bufo bufo* populations through time, based on the Bayesian phylogeographical analyses implemented in Beast and carried out independently on the mtDNA dataset. Four time intervals are shown, and a full representation of the diffusion process is provided as Supplementary File S1 information. Polygons represent 80% HPD uncertainty for the spatial location of ancestral populations. Time is in thousand years before present (ka). Images obtained from GOOGLE EARTH PRO 7.3.3 (https://www.google.com/earth/versions/#earth-pro) and modified using the software Canvas 11 (ACD Systems of America, Inc.).

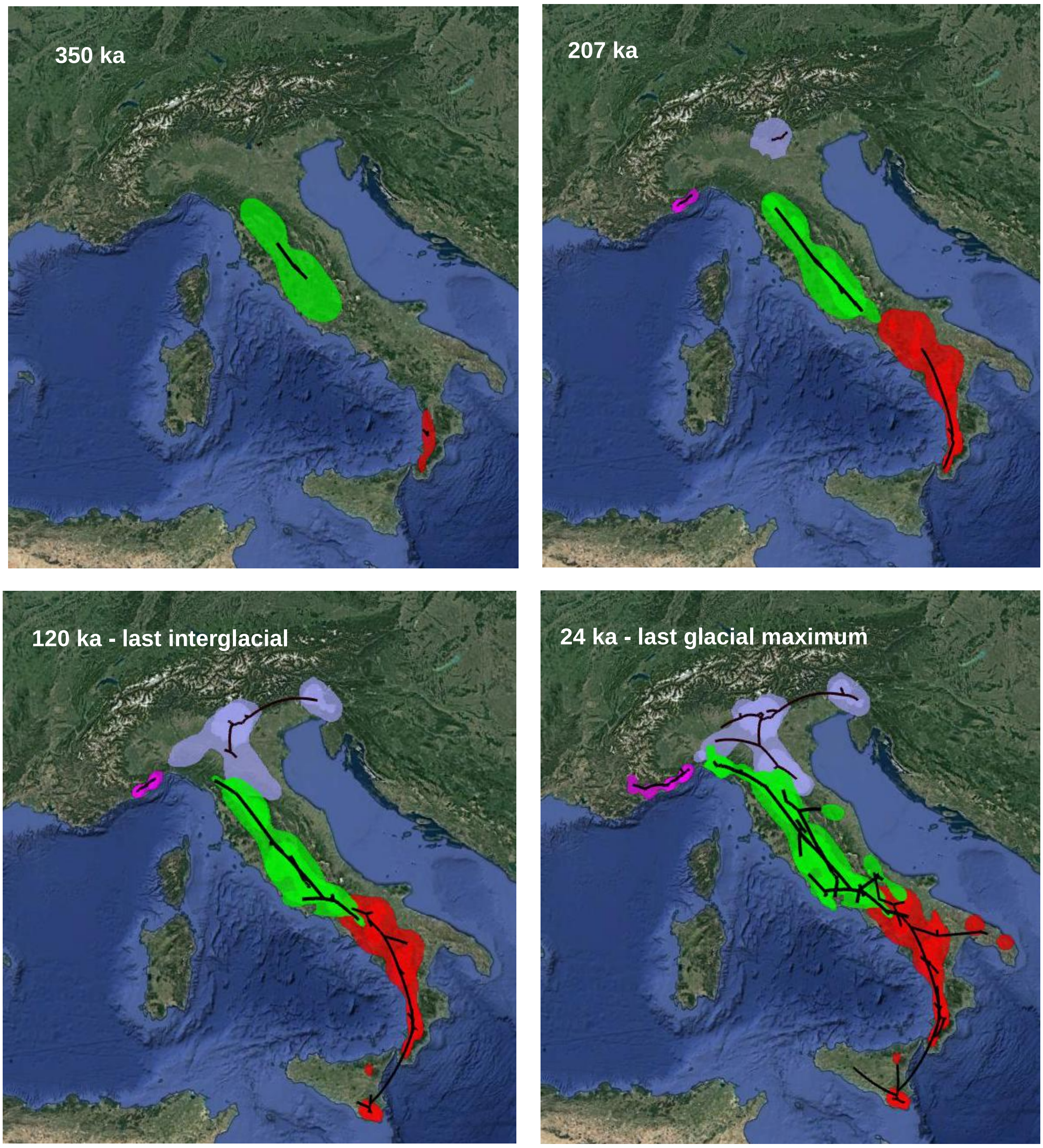

Supplement: Supplementary file 3 — Supplementary Figure S3. [file 41598_2020_79046_MOESM3_ESM.pdf]
