# Supplementary material for: Reconstructing hotspots of genetic diversity from glacial refugia and subsequent dispersal in Italian common toads (Bufo bufo)
Source: Sci Rep. 2021 Jan 8;11:260. doi: 10.1038/s41598-020-79046-y (PMC7794404; doi:10.1038/s41598-020-79046-y)
Supplement: Supplementary file 5 — Supplementary Table S1. [file 41598_2020_79046_MOESM5_ESM.pdf]

Scientific Reports

SUPPORTING INFORMATION

Reconstructing hotspots of genetic diversity from glacial refugia and subsequent dispersal in Italian common toads (*Bufo bufo*)

Andrea Chiocchio, Jan. W. Arntzen, Iñigo Martínez-Solano, Wouter de Vries, Roberta Bisconti, Alice Pezzarossa, Luigi Maiorano, Daniele Canestrelli

Supplementary Table S1 – GENBANK accession for each studied individual.

Table S1 - GENBANK accession numbers for all the mitochondrial DNA sequences of the common toads analysed for the present study.

Sample	Location	Lat	Long	individual	Genbank accessions	
					Cytb	16s
1	Godz	45.898	13.989	1.1	MW350662	MW350431
1	Godz	45.898	13.989	1.2	MW350663	MW350432
1	Godz	45.898	13.989	1.3	MW350664	MW350433
1	Godz	45.898	13.989	1.4	MW350665	MW350434
1	Godz	45.898	13.989	1.5	MW350666	MW350435
2	Musi	46.316	13.251	2.1	MW350523	MW350292
2	Musi	46.316	13.251	2.2	MW350524	MW350293
2	Musi	46.316	13.251	2.3	MW350525	MW350294
5	Monte Baldo	45.678	10.780	5.1	MW350471	MW350240
5	Monte Baldo	45.678	10.780	5.2	MW350472	MW350241
5	Monte Baldo	45.678	10.780	5.3	MW350473	MW350242
5	Monte Baldo	45.678	10.780	5.4	MW350474	MW350243
6	Endine Gaiano	45.792	10.009	6.1	MW350459	MW350228
7	Arona	45.734	8.552	7.1	MW350462	MW350231
8	Borgofranco di Ivrea	45.511	7.873	8.1	MW350461	MW350230
11	Barge	44.716	7.326	11.1	MW350460	MW350229
13	Thorame Haute	44.089	6.534	13.1	MW350681	MW350450
13	Thorame Haute	44.089	6.534	13.2	MW350682	MW350451
13	Thorame Haute	44.089	6.534	13.3	MW350683	MW350452
13	Thorame Haute	44.089	6.534	13.4	MW350684	MW350453
14	Saint-Auban	43.843	6.727	14.1	MW350647	MW350416
14	Saint-Auban	43.843	6.727	14.2	MW350648	MW350417
14	Saint-Auban	43.843	6.727	14.3	MW350649	MW350418
14	Saint-Auban	43.843	6.727	14.4	MW350650	MW350419
14	Saint-Auban	43.843	6.727	14.5	MW350651	MW350420
15	La Martre	43.797	6.598	15.1	MW350667	MW350436
15	La Martre	43.797	6.598	15.2	MW350668	MW350437
15	La Martre	43.797	6.598	15.3	MW350669	MW350438
15	La Martre	43.797	6.598	15.4	MW350670	MW350439
15	La Martre	43.797	6.598	15.5	MW350671	MW350440
16	Gattières	43.759	7.174	16.1	MW350657	MW350426

Sample	Location	Lat	Long	individual	Genbank accessions	
					Cytb	16s
16	Gattières	43.759	7.174	16.2	MW350658	MW350427
16	Gattières	43.759	7.174	16.3	MW350659	MW350428
16	Gattières	43.759	7.174	16.4	MW350660	MW350429
16	Gattières	43.759	7.174	16.5	MW350661	MW350430
17	Rocchetta Nervina	43.883	7.603	17.1	MW350526	MW350295
17	Rocchetta Nervina	43.883	7.603	17.2	MW350527	MW350296
17	Rocchetta Nervina	43.883	7.603	17.3	MW350528	MW350297
17	Rocchetta Nervina	43.883	7.603	17.4	MW350529	MW350298
17	Rocchetta Nervina	43.883	7.603	17.5	MW350530	MW350299
18	Ceriana_a	43.880	7.773	18.1	MW350627	MW350396
18	Ceriana_a	43.880	7.773	18.2	MW350628	MW350397
18	Ceriana_a	43.880	7.773	18.3	MW350629	MW350398
18	Ceriana_a	43.880	7.773	18.4	MW350630	MW350399
18	Ceriana_a	43.880	7.773	18.5	MW350631	MW350400
18	Ceriana_a	43.880	7.773	18.6	MW350632	MW350401
18	Ceriana_a	43.880	7.773	18.7	MW350633	MW350402
18	Ceriana_a	43.880	7.773	18.8	MW350634	MW350403
18	Ceriana_a	43.880	7.773	18.9	MW350635	MW350404
19	Molini di Triora	43.988	7.776	19.1	MW350636	MW350405
19	Molini di Triora	43.988	7.776	19.2	MW350637	MW350406
19	Molini di Triora	43.988	7.776	19.3	MW350638	MW350407
20	Mendatica	44.072	7.811	20.1	MW350639	MW350408
20	Mendatica	44.072	7.811	20.2	MW350640	MW350409
20	Mendatica	44.072	7.811	20.3	MW350641	MW350410
21	Lecchiore	43.916	7.921	21.1	MW350507	MW350276
21	Lecchiore	43.916	7.921	21.2	MW350508	MW350277
21	Lecchiore	43.916	7.921	21.3	MW350509	MW350278
21	Lecchiore	43.916	7.921	21.4	MW350510	MW350279
21	Lecchiore	43.916	7.921	21.5	MW350511	MW350280
21	Lecchiore	43.916	7.921	21.6	MW350465	MW350234
23	Calice Ligure	44.203	8.287	23.1	MW350652	MW350421
23	Calice Ligure	44.203	8.287	23.2	MW350653	MW350422
23	Calice Ligure	44.203	8.287	23.3	MW350654	MW350423
24	Albisola Superiore	44.343	8.496	24.1	MW350642	MW350411
24	Albisola Superiore	44.343	8.496	24.2	MW350643	MW350412
24	Albisola Superiore	44.343	8.496	24.3	MW350644	MW350413
24	Albisola Superiore	44.343	8.496	24.4	MW350645	MW350414
24	Albisola Superiore	44.343	8.496	24.5	MW350646	MW350415
25	Brignano-Frascata	44.829	9.038	25.1	MW350463	MW350232
26	Lerma	44.620	8.712	26.1	MW350516	MW350285
26	Lerma	44.620	8.712	26.2	MW350517	MW350286
26	Lerma	44.620	8.712	26.3	MW350518	MW350287
26	Lerma	44.620	8.712	26.4	MW350519	MW350288
27	San Giorgio a Bavari	44.428	9.011	27.1	MW350466	MW350235
28	Zavattarello	44.891	9.264	28.1	MW350467	MW350236
29	Varese Ligure	44.480	9.607	29.1	MW350566	MW350335
29	Varese Ligure	44.480	9.607	29.2	MW350567	MW350336

Sample	Location	Lat	Long	individual	Genbank accessions	
					Cytb	16s
29	Varese Ligure	44.480	9.607	29.3	MW350568	MW350337
30	Sarzana Ligure	44.270	9.458	30.1	MW350464	MW350233
31	Cerreto Laghi	44.303	10.244	31.1	MW350486	MW350255
31	Cerreto Laghi	44.303	10.244	31.2	MW350487	MW350256
32	Monte San Pietro	44.360	11.108	32.1	MW350454	MW350223
33	Monghidoro	44.248	11.346	33.1	MW350578	MW350347
33	Monghidoro	44.248	11.346	33.2	MW350579	MW350348
34	Campo Tizzoro	44.039	10.862	34.1	MW350593	MW350362
34	Campo Tizzoro	44.039	10.862	34.2	MW350594	MW350363
34	Campo Tizzoro	44.039	10.862	34.3	MW350595	MW350364
35	Ciola	43.983	12.130	35.1	MW350455	MW350224
36	Terranuova Bracciolini	43.555	11.569	36.1	MW350555	MW350324
36	Terranuova Bracciolini	43.555	11.569	36.2	MW350556	MW350325
36	Terranuova Bracciolini	43.555	11.569	36.3	MW350557	MW350326
36	Terranuova Bracciolini	43.555	11.569	36.4	MW350558	MW350327
36	Terranuova Bracciolini	43.555	11.569	36.5	MW350559	MW350328
36	Terranuova Bracciolini	43.555	11.569	36.6	MW350458	MW350227
37	Serra San Quirico	43.428	13.044	37.1	MW350456	MW350225
39	San Gemini	42.608	12.558	39.1	MW350545	MW350314
39	San Gemini	42.608	12.558	39.2	MW350546	MW350315
39	San Gemini	42.608	12.558	39.3	MW350547	MW350316
39	San Gemini	42.608	12.558	39.4	MW350548	MW350317
39	San Gemini	42.608	12.558	39.5	MW350549	MW350318
40	Canale Monterano	42.140	12.097	40.1	MW350569	MW350338
40	Canale Monterano	42.140	12.097	40.2	MW350570	MW350339
40	Canale Monterano	42.140	12.097	40.3	MW350587	MW350356
40	Canale Monterano	42.140	12.097	40.4	MW350588	MW350357
40	Canale Monterano	42.140	12.097	40.5	MW350589	MW350358
40	Canale Monterano	42.140	12.097	40.6	MW350590	MW350359
41	Rocca Sinibalda	42.275	12.926	41.1	MW350596	MW350365
41	Rocca Sinibalda	42.275	12.926	41.2	MW350597	MW350366
41	Rocca Sinibalda	42.275	12.926	41.3	MW350598	MW350367
42	Scoppito	42.361	13.265	42.1	MW350457	MW350226
43	Jenne	41.890	13.171	43.1	MW350580	MW350349
43	Jenne	41.890	13.171	43.2	MW350581	MW350350
43	Jenne	41.890	13.171	43.3	MW350582	MW350351
43	Jenne	41.890	13.171	43.4	MW350591	MW350360
43	Jenne	41.890	13.171	43.5	MW350592	MW350361
44	Bosco del Foglino	41.471	12.719	44.1	MW350575	MW350344
44	Bosco del Foglino	41.471	12.719	44.2	MW350576	MW350345
44	Bosco del Foglino	41.471	12.719	44.3	MW350577	MW350346
45	Fara Filorum Petri	42.248	14.188	45.1	MW350494	MW350263
45	Fara Filorum Petri	42.248	14.188	45.2	MW350495	MW350264
45	Fara Filorum Petri	42.248	14.188	45.3	MW350496	MW350265
46	Opi	41.791	13.807	46.1	MW350531	MW350300
46	Opi	41.791	13.807	46.2	MW350532	MW350301
46	Opi	41.791	13.807	46.3	MW350533	MW350302

Sample	Location	Lat	Long	individual	Genbank accessions	
					Cytb	16s
47	Doganella	41.750	12.761	47.1	MW350571	MW350340
47	Doganella	41.750	12.761	47.2	MW350572	MW350341
47	Doganella	41.750	12.761	47.3	MW350573	MW350342
47	Doganella	41.750	12.761	47.4	MW350574	MW350343
48	Molella	41.268	13.046	48.1	MW350583	MW350352
48	Molella	41.268	13.046	48.2	MW350584	MW350353
48	Molella	41.268	13.046	48.3	MW350585	MW350354
48	Molella	41.268	13.046	48.4	MW350586	MW350355
49	San Pietro Infine	41.444	13.968	49.1	MW350500	MW350269
49	San Pietro Infine	41.444	13.968	49.2	MW350501	MW350270
49	San Pietro Infine	41.444	13.968	49.3	MW350502	MW350271
50	San marco la Catola	41.541	15.040	50.1	MW350550	MW350319
50	San marco la Catola	41.541	15.040	50-2	MW350551	MW350320
50	San marco la Catola	41.541	15.040	50.3	MW350552	MW350321
51	Lago Matese	41.409	14.405	51.1	MW350520	MW350289
51	Lago Matese	41.409	14.405	51.2	MW350521	MW350290
51	Lago Matese	41.409	14.405	51.3	MW350522	MW350291
51	Lago Matese	41.409	14.405	51.4	MW350672	MW350441
51	Lago Matese	41.409	14.405	51.5	MW350673	MW350442
52	Biccari	41.370	15.172	52.1	MW350475	MW350244
52	Biccari	41.370	15.172	52.2	MW350476	MW350245
52	Biccari	41.370	15.172	52.3	MW350477	MW350246
53	Grata	41.276	13.710	53.1	MW350497	MW350266
53	Grata	41.276	13.710	53.2	MW350498	MW350267
53	Grata	41.276	13.710	53.3	MW350499	MW350268
54	Camposauro	41.174	14.582	54.1	MW350542	MW350311
54	Camposauro	41.174	14.582	54.2	MW350543	MW350312
54	Camposauro	41.174	14.582	54.3	MW350544	MW350313
54	Camposauro	41.174	14.582	54.4	MW350679	MW350448
54	Camposauro	41.174	14.582	54.5	MW350680	MW350449
55	Tufara	41.061	14.714	55.1	MW350563	MW350332
55	Tufara	41.061	14.714	55.2	MW350564	MW350333
55	Tufara	41.061	14.714	55.3	MW350565	MW350334
56	Spinazzola	40.998	16.059	56.1	MW350553	MW350322
56	Spinazzola	40.998	16.059	56.2	MW350554	MW350323
57	Monticchio	40.929	15.603	57.1	MW350483	MW350252
57	Monticchio	40.929	15.603	57.2	MW350484	MW350253
57	Monticchio	40.929	15.603	57.3	MW350485	MW350254
57	Monticchio	40.929	15.603	57.4	MW350655	MW350424
57	Monticchio	40.929	15.603	57.5	MW350656	MW350425
58	Lago Laceno	40.806	15.095	58.1	MW350503	MW350272
58	Lago Laceno	40.806	15.095	58.2	MW350504	MW350273
58	Lago Laceno	40.806	15.095	58.3	MW350505	MW350274
58	Lago Laceno	40.806	15.095	58.4	MW350506	MW350275
58	Lago Laceno	40.806	15.095	58.5	MW350623	MW350392
58	Lago Laceno	40.806	15.095	58.6	MW350624	MW350393
58	Lago Laceno	40.806	15.095	58.7	MW350625	MW350394

Sample	Location	Lat	Long	individual	Genbank accessions	
					Cytb	16s
58	Lago Laceno	40.806	15.095	58.8	MW350626	MW350395
59	Tricarico	40.618	16.145	59.1	MW350560	MW350329
59	Tricarico	40.618	16.145	59.2	MW350561	MW350330
59	Tricarico	40.618	16.145	59.3	MW350562	MW350331
60	Campora	40.289	15.327	60.1	MW350539	MW350308
60	Campora	40.289	15.327	60.2	MW350540	MW350309
60	Campora	40.289	15.327	60.3	MW350541	MW350310
61	Lago Cessuta	40.254	15.784	61.1	MW350478	MW350247
61	Lago Cessuta	40.254	15.784	61.2	MW350479	MW350248
61	Lago Cessuta	40.254	15.784	61.3	MW350480	MW350249
61	Lago Cessuta	40.254	15.784	61.4	MW350481	MW350250
61	Lago Cessuta	40.254	15.784	61.5	MW350482	MW350251
62	Contrada Massadita	39.942	15.806	62.1	MW350611	MW350380
62	Contrada Massadita	39.942	15.806	62.2	MW350612	MW350381
62	Contrada Massadita	39.942	15.806	62.3	MW350613	MW350382
62	Contrada Massadita	39.942	15.806	62.4	MW350614	MW350383
63	Orsomarso	39.800	15.908	63.1	MW350615	MW350384
63	Orsomarso	39.800	15.908	63.2	MW350616	MW350385
63	Orsomarso	39.800	15.908	63.3	MW350617	MW350386
64	Lago Farneto	39.664	16.157	64.1	MW350489	MW350258
64	Lago Farneto	39.664	16.157	64.2	MW350490	MW350259
64	Lago Farneto	39.664	16.157	64.3	MW350491	MW350260
64	Lago Farneto	39.664	16.157	64.4	MW350492	MW350261
64	Lago Farneto	39.664	16.157	64.5	MW350493	MW350262
65	Fagnano Castello	39.556	16.021	65.1	MW350603	MW350372
65	Fagnano Castello	39.556	16.021	65.2	MW350604	MW350373
67	Fiumefreddo Bruzio	39.225	16.072	67.1	MW350602	MW350371
67	Fiumefreddo Bruzio	39.225	16.072	67.2	MW350605	MW350374
67	Fiumefreddo Bruzio	39.225	16.072	67.3	MW350606	MW350375
67	Fiumefreddo Bruzio	39.225	16.072	67.4	MW350607	MW350376
67	Fiumefreddo Bruzio	39.225	16.072	67.5	MW350608	MW350377
68	Falerna	39.002	16.174	68.1	MW350488	MW350257
69	Lago dell'Angitola	38.740	16.236	69.1	MW350600	MW350369
69	Lago dell'Angitola	38.740	16.236	69.2	MW350601	MW350370
70	Stilo	38.478	16.469	70.1	MW350622	MW350391
71	Oppido Mamertino	38.291	15.989	71.1	MW350534	MW350303
71	Oppido Mamertino	38.291	15.989	71.2	MW350535	MW350304
71	Oppido Mamertino	38.291	15.989	71.3	MW350536	MW350305
71	Oppido Mamertino	38.291	15.989	71.4	MW350537	MW350306
71	Oppido Mamertino	38.291	15.989	71.5	MW350538	MW350307
72	Gambarie	38.181	15.846	72.1	MW350609	MW350378
72	Gambarie	38.181	15.846	72.2	MW350610	MW350379
73	Alberobello	40.780	17.254	73.1	MW350470	MW350239
73	Alberobello	40.780	17.254	73.2	MW350599	MW350368
74	Lecce	40.345	18.167	74.1	MW350674	MW350443
74	Lecce	40.345	18.167	74.2	MW350675	MW350444
74	Lecce	40.345	18.167	74.3	MW350676	MW350445

Sample	Location	Lat	Long	individual	Genbank accessions	
					Cytb	16s
74	Lecce	40.345	18.167	74.4	MW350677	MW350446
74	Lecce	40.345	18.167	74.5	MW350678	MW350447
75	Fiume Irminio	36.929	14.674	75.1	MW350618	MW350387
75	Fiume Irminio	36.929	14.674	75.2	MW350619	MW350388
75	Fiume Irminio	36.929	14.674	75.3	MW350620	MW350389
75	Fiume Irminio	36.929	14.674	75.4	MW350621	MW350390
76	Corleone	37.869	13.305	76.1	MW350512	MW350281
76	Corleone	37.869	13.305	76.2	MW350513	MW350282
76	Corleone	37.869	13.305	76.3	MW350514	MW350283
76	Corleone	37.869	13.305	76.4	MW350515	MW350284
77	Rosolini	36.823	15.032	77.1	MW350469	MW350238
78	Maletto	37.853	14.831	78.1	MW350468	MW350237
