# Supplementary material for: Reconstructing hotspots of genetic diversity from glacial refugia and subsequent dispersal in Italian common toads (Bufo bufo)
Source: Sci Rep. 2021 Jan 8;11:260. doi: 10.1038/s41598-020-79046-y (PMC7794404; doi:10.1038/s41598-020-79046-y)
Supplement: Supplementary file 7 — Supplementary Table S3. [file 41598_2020_79046_MOESM7_ESM.pdf]

Scientific Reports

SUPPORTING INFORMATION

Reconstructing hotspots of genetic diversity from glacial refugia and subsequent dispersal in Italian common toads (*Bufo bufo*)

Andrea Chiocchio, Jan. W. Arntzen, Iñigo Martínez-Solano, Wouter de Vries, Roberta Bisconti, Alice Pezzarossa, Luigi Maiorano, Daniele Canestrelli

Appendix S3 – Performances of the SDM analyses

Supplementary Table S3. Performance of the species distribution modeling analyses for the central and southern lineages of *B. bufo*: a) AUC values for each algorithm and for each of the five modelling runs; b) importance of variables for each algorithm, averaged across all the runs (values ranging from 0 to 1, with 0 = no importance and 1 = maximum importance)

a)

	GLM	GBM	GAM	MARS	MAXENT
central	0.755	0.832	0.801	0.773	0.796
southern	0.753	0.898	0.803	0.783	0.813
central	0.755	0.832	0.804	0.777	0.797
southern	0.758	0.893	0.803	0.788	0.81
central	0.756	0.832	0.800	0.774	0.796
southern	0.757	0.888	0.795	0.781	0.803
central	0.754	0.833	0.800	0.773	0.797
southern	0.752	0.887	0.804	0.788	0.808
central	0.754	0.829	0.799	0.774	0.794
southern	0.756	0.889	0.802	0.779	0.803

b)

**Central lineage**	GLM	GBM	GAM	MARS	MAXENT
Annual mean temperature	0.006	0.002	0.408	0.172	0.031
Mean diurnal range	0.010	0.026	0.053	0.002	0.011
Temperature seasonality	0.295	0.213	0.297	0.216	0.232
Mean temperature of wettest quarter	0.067	0.056	0.123	0.037	0.045
Mean temperature of driest quarter	0.230	0.138	0.570	0.250	0.228
Precipitation of wettest month	0.197	0.178	0.196	0.199	0.204
Precipitation sasonality	0.534	0.303	0.439	0.514	0.543

**Southern lineage**	GLM	GBM	GAM	MARS	MAXENT
Mean diurnal range	0.010	0.118	0.034	0.000	0.039
Temperature seasonality	0.100	0.138	0.277	0.149	0.186
Mean temperature of wettest quarter	0.061	0.029	0.139	0.055	0.012
Mean temperature of driest quarter	0.130	0.050	0.286	0.284	0.100
Precipitation of warmest quarter	0.750	0.574	0.576	0.750	0.705
Precipitation of coldest quarter	0.007	0.082	0.056	0.011	0.033
